# Diagnostic and Therapeutic Strategies for Fluoropyrimidine Treatment of Patients Carrying Multiple *DPYD* Variants

**DOI:** 10.3390/genes9120585

**Published:** 2018-11-28

**Authors:** Carin A. T. C. Lunenburg, Linda M. Henricks, André B. P. van Kuilenburg, Ron H. J. Mathijssen, Jan H. M. Schellens, Hans Gelderblom, Henk-Jan Guchelaar, Jesse J. Swen

**Affiliations:** 1 Department of Medical Oncology, Leiden University Medical Center, 2333 ZA Leiden, The Netherlands; c.a.t.c.lunenburg@lumc.nl (C.A.T.C.L.); a.j.gelderblom@lumc.nl (H.G.); 2 Department of Clinical Pharmacology, Division of Medical Oncology, The Netherlands Cancer Institute, 1066 CX Amsterdam, The Netherlands; l.henricks@nki.nl (L.M.H.); j.schellens@gmail.com (J.H.M.S.); 3 Division of Pharmacology, The Netherlands Cancer Institute, 1066 CX Amsterdam, The Netherlands; 4 Department of Clinical Chemistry, Amsterdam University Medical Centre, 1105 AZ Amsterdam, The Netherlands; a.b.vankuilenburg@amc.uva.nl; 5 Department of Medical Oncology, Erasmus MC Cancer Institute, 3015 GD Rotterdam, The Netherlands; a.mathijssen@erasmusmc.nl; 6 Department of Clinical Pharmacy & Toxicology, Leiden University Medical Center, 2333 ZA Leiden, The Netherlands; h.j.guchelaar@lumc.nl

**Keywords:** pharmacogenomics, pharmacogenetics, genotype, phenotype, alleles, precision medicine

## Abstract

*DPYD* genotyping prior to fluoropyrimidine treatment is increasingly implemented in clinical care. Without phasing information (i.e., allelic location of variants), current genotype-based dosing guidelines cannot be applied to patients carrying multiple *DPYD* variants. The primary aim of this study is to examine diagnostic and therapeutic strategies for fluoropyrimidine treatment of patients carrying multiple *DPYD* variants. A case series of patients carrying multiple *DPYD* variants is presented. Different genotyping techniques were used to determine phasing information. Phenotyping was performed by dihydropyrimidine dehydrogenase (DPD) enzyme activity measurements. Publicly available databases were queried to explore the frequency and phasing of variants of patients carrying multiple *DPYD* variants. Four out of seven patients carrying multiple *DPYD* variants received a full dose of fluoropyrimidines and experienced severe toxicity. Phasing information could be retrieved for four patients. In three patients, variants were located on two different alleles, i.e., in *trans*. Recommended dose reductions based on the phased genotype differed from the phenotype-derived dose reductions in three out of four cases. Data from publicly available databases show that the frequency of patients carrying multiple *DPYD* variants is low (< 0.2%), but higher than the frequency of the commonly tested DPYD*13 variant (0.1%). Patients carrying multiple *DPYD* variants are at high risk of developing severe toxicity. Additional analyses are required to determine the correct dose of fluoropyrimidine treatment. In patients carrying multiple *DPYD* variants, we recommend that a DPD phenotyping assay be carried out to determine a safe starting dose.

## 1. Introduction

Fluoropyrimidines (including 5-fluorouracil (5-FU) and capecitabine) are the cornerstone of treatment for various types of cancer and are used by millions of patients worldwide each year [1,2,3]. However, up to one-third of treated patients experience severe toxicity (Common Terminology Criteria for Adverse Events (CTC-AE) grade ≥ 3), such as diarrhea, hand–foot syndrome, or mucositis upon treatment with fluoropyrimidines [4,5]. These adverse events can lead to mortality in approximately 1% of patients who experience severe toxicity [4,6]. Dihydropyrimidine dehydrogenase (DPD) is the key enzyme in the metabolism of 5-FU and its decreased activity is strongly associated with toxicity [7,8]. Variants in *DPYD*, the gene encoding DPD, can lead to decreased DPD enzyme activity [9,10,11,12]. Prospective *DPYD* genotyping of four main *DPYD* variants followed by dose reductions in patients carrying any of these four *DPYD* variants is safe, cost-effective, and feasible in clinical practice [13,14,15]. These *DPYD* variants are DPYD*2A (rs3918290, c.1905+1G>A, IVS14+1G>A); DPYD*13 (rs55886062, c.1679T>G, I560S); c.1236G>A/HapB3 (rs56038477, E412E); and c.2846A>T (rs67376798, D949V). For these four variants, convincing evidence has been provided warranting implementation in clinical practice [4,5,12,15,16,17].

An increasing number of hospitals apply prospective *DPYD* genotyping when treating patients with fluoropyrimidines [18]. Individual dosing guidelines for the abovementioned four *DPYD* variants are provided by the Dutch Pharmacogenetics Working Group (DPWG) and the Clinical Pharmacogenetics Implementation Consortium (CPIC) [19,20]. Dosing guidelines are based on the expected remaining DPD enzyme activity and can be applied to patients who are heterozygous carriers of a single *DPYD* variant. For homozygous *DPYD* variant allele carriers (two identical variants) and compound heterozygous *DPYD* variant allele carriers (two or more different variants), dosing guidelines are not yet available (or treatment with an alternative drug is advised), although safe treatment with low-dose fluoropyrimidines in these homozygous *DPYD* patients was demonstrated by a recent case series [21].

**Figure 1:**
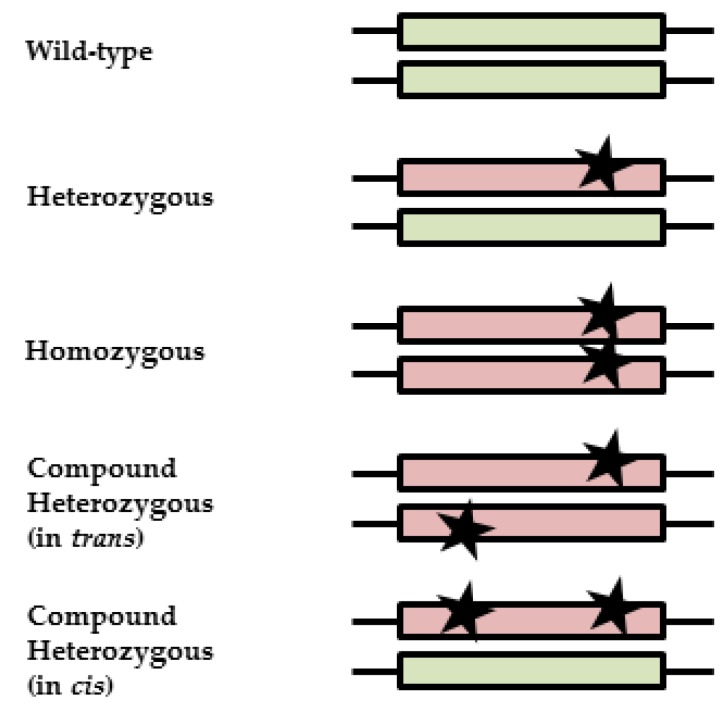
Illustration of zygosity and clinical interpretations. Black stars represent variants; boxes represent alleles. A wild-type patient carries no variants, resulting in normal-activity alleles (green). A heterozygous patient carries one variant, resulting in one reduced or inactive allele (red) and one active allele (green). A partly reduced enzyme activity is expected, since there is still one active allele left. For homozygous patients, both variants result in a reduced or inactive allele (red). Depending on the effect of the variants on the protein, a reduced or absent enzyme activity is expected. Compound heterozygous patients can carry variants on different alleles (in *trans*) or on one allele (in *cis*), resulting in differences in enzyme function, either like that of a heterozygous patient or a homozygous patient.

Patients who carry multiple variants (compound heterozygous) can carry the variants on a single allele (in *cis*) or on different alleles (in *trans*). In the first case, one functionally active allele remains, whereas in the latter case, both alleles are affected, which may result in a proportionally decreased enzyme activity (Figure 1). With currently used genotyping techniques, the allelic location of variants (phasing) cannot be determined. This uncertainty hampers adequate interpretation of the pharmacogenetic test result in compound heterozygous patients and makes it impossible to give an appropriate dose recommendation based on the genotype alone. Since it is likely that in the future, even more *DPYD* variants will be tested, the probability of finding compound heterozygous *DPYD* variant allele carriers will increase. The aims of this study are to examine diagnostic and therapeutic strategies for fluoropyrimidine treatment of patients carrying multiple *DPYD* variants and the frequency and phasing of variants of compound heterozygous *DPYD* patients in publicly available databases.

## 2. Materials and Methods

In this study, we present seven compounds heterozygous *DPYD* variant allele carriers as clinical cases. In addition, we have performed in silico research in publicly available databases.

### 2.1. Patients

Data and DNA from patient cases carrying multiple *DPYD* variants were collected. Patients were identified either after development of severe toxicity from fluoropyrimidine-containing therapy, by additional retrospective genotyping in a clinical trial (clinicaltrials.gov identifier NCT00838370, [13]), or prior to treatment in routine clinical care. The study was reviewed and approved by the institutional review board of the Leiden University Medical Centre (LUMC, G18.015). Patient data from the electronic medical records was handled following the codes of proper use and proper conduct in the self-regulatory codes of conduct [22]. Toxicity to fluoropyrimidine-containing therapy was graded by the treating physicians using the National Cancer Institute CTC-AE version 4.03 [23], and severe fluoropyrimidine-induced toxicity was defined as CTC-AE grade ≥ 3. In some cases, additional patient material to determine the phasing of the *DPYD* variants was collected. In these cases, additional patient consent was obtained.

### 2.2. Dihydropyrimidine Dehydrogenase Enzyme Activity Measurements

For all patients, DPD enzyme activity was determined. This could be either prior to treatment or retrospectively after the occurrence of severe toxicity. DPD enzyme activity measurement in peripheral blood mononuclear cells (PBMCs) [24,25] was used as a reference to assess DPD activity, and has been used previously to determine dosages in *DPYD* variant-carrying patients [21,26]. A validated method [27] was used, containing radiolabeled thymine as a substrate and consisting of high-performance liquid chromatography (HPLC) with online radioisotope detection using liquid scintillation counting. Normal values for healthy volunteers are 9.9 ± 2.8 nmol/(mg×h), for DPD-deficient patients are 4.8 ± 1.7 nmol/(mg×h), and reference values range from 5.9 to 14 nmol/(mg×h) [28]. Dose reductions based on DPD enzyme activity were performed in a one-to-one ratio, as was previously described by Henricks et al. [21]. Thereafter, toxicity-guided dosing was used.

### 2.3. Molecular Methods for Estimation of Phasing

In regard to the size of the *DPYD* gene, the location of the variants, and the material available (DNA, RNA) from the patients, three molecular methods to determine the phasing of the variants could be used in this study. In four patients, we could execute one or more of these methods. These methods are explained and illustrated in the supplementary material (Figure S1). Details on these techniques have been published elsewhere [29,30,31].

### 2.4. Frequencies of Compound Heterozygous DPYD Carriers

To investigate the incidence of compound heterozygous *DPYD* variant allele carriers (of the four genotyped *DPYD* variants), large databases were queried [32,33]. The incidence was calculated using minor allele frequencies (MAFs) of each variant identified in the databases separately. Since the determined variants are not in the same haplotype, it was assumed that the inheritance of these individual *DPYD* variants is independent. All genotypes from the databases were calculated to be in Hardy–Weinberg equilibrium, except for DPYD*2A and c.1236G>A for the Exome Aggregation Consortium (ExAC, http://exac.broadinstitute.org/) [32] and Genome Aggregation Database (gnomAD, http://gnomad.broadinstitute.org/) [33] due to a slight overrepresentation of homozygous cases. The calculated frequencies were compared to frequencies from databases in which phasing could be determined.

#### Exome Aggregation Consortium and Genome Aggregation Database

Both the ExAC [32] and gnomAD [33] databases collect exome sequencing data and aggregate the data for public use. The ExAC dataset (v0.3.1) contains sequenced data of 60,706 unrelated individuals. The gnomAD dataset (v2.0) contains sequenced data of 123,136 exomes and 15,496 genomes from unrelated individuals. In ExAC, 2791 *DPYD* variants, and in gnomAD, 2190 *DPYD* variants were found. MAFs of *DPYD* variants from these databases reflect those of the population due to the large group size in the databases. Since both ExAC and gnomAD do not contain individual matched or phased data, it is not possible to search for compound heterozygous patients in these databases.

### 2.5. Phasing in Compound Heterozygous DPYD Carriers

Three databases were used to identify compound heterozygous *DPYD* variant allele carriers and determine the phasing, i.e., allelic location, of variants.

#### 2.5.1. Genome of the Netherlands Datasets

The Genome of the Netherlands (GoNL, http://www.nlgenome.nl/) trio datasets contain information of related fathers, mothers, and children, and phasing information is therefore available. Datasets were previously processed and phased using trio-aware variant calling [34]. After the exclusion of children, phased variant call format (VCF) files for 496 subjects (fathers and mothers) were obtained from the GoNL repository. The toolset Bedtools (https://bedtools.readthedocs.io/en/latest/, v2) was used to extract all variants found in the *DPYD* locus (chr1:97,543,300–98,386,615). Next, for all individuals, the carrier status of DPYD*2A, DPYD*13, c.1236G>A, and c.2846A>T was examined. Individuals who carry at least one of the four actionable *DPYD* variants were identified, and using a custom Python [35] script, the phasing of variants was assessed for individuals with multiple variants.

#### 2.5.2. 1000 Genomes Database

The 1000 Genomes Project (http://www.internationalgenome.org/) is the largest publicly available catalogue of human variation and genotyped phased data. It originally ran from 2008 until 2015, and thereafter it was maintained and expanded by the International Genome Sample Resource (IGSR) [36]. On 27 October 2016, phased data of the *DPYD* gene (chr1: 97,543,300–98,386,615) was downloaded from the 1000 Genomes ftp server (phase 3; GRCh37; chr1: 97,543,300–98,386,615) using Tabix (v1.1) [37]. The statistical program R (v3.2.5) [38] was used to select the genotypes at four *DPYD* risk alleles in unrelated individuals of Caucasian descent.

#### 2.5.3. Exome Trios Leiden University Medical Centre Database

This diagnostic database of the clinical genetics department of LUMC contains 433 complete exome trios (father, mother, and child). The exome was enriched by the Agilent sureselect v5 kit and sequenced using various Illumina (San Diego, CA, USA) sequencers (Hiseq 2000, 2500, 4000, Nextseq). Carrier status of the abovementioned *DPYD* variants was established by querying the trio VCF files. We also investigated all samples with sufficient coverage of this region to obtain a reliable frequency estimate. In the case of trios, only parents were taken into account.

## 3. Results

### 3.1. Patient Cases and Clinical Implications

Details of the demographics and clinical characteristics of the seven cases are described in the supplementary material (patient cases). All patients received treatment with fluoropyrimidines and were identified as compound heterozygous *DPYD* variant allele carriers either prior to the start of treatment or retrospectively. Table 1 shows an overview of the cases. Table 2 shows all genotype and phenotype results. With additional genetic testing, phasing could be determined in four out of seven patients. In three patients, the variants were located in *trans*, and one patient carried the variants in *cis*. With the phasing information available, it is possible to calculate a dose recommendation using publicly available pharmacogenetic dosing guidelines [19,20]. For example, patient 1 carried DPYD*2A and c.1236G>A in *trans*. The gene activity values range from inactive (0) to fully active (1). DPYD*2A and c.1236G>A/HapB3 have values of 0 and 0.5, respectively. As this patient carries the variants in *trans*, each allele contains one variant and no fully functional allele remains. Therefore, the cumulated gene activity score (GAS) is 0.5. The GAS can be used to determine dose recommendations according to the genotype, as was previously described [12]. The GAS ranges from 0 to 2, and a score of 0.5 corresponds to a dose recommendation of 25%. The DPD enzyme activity of patient 1 was 0.9 nmol/(mg×h). This was divided by the mean of the reference value (9.9), which results in a theoretical DPD activity of 9%. For each patient for whom phasing details were known, the GAS was determined and compared to the theoretical DPD activity. Dose recommendations according to the GAS (genotype) and theoretical DPD activity (phenotype) were divergent in almost all cases, as shown in Table 2.

### 3.2. Preventing Toxicity

Three of the seven case patients were identified as carriers of one or more *DPYD* variants prior to the start of therapy. For one patient, the DPD enzyme activity was determined prior to the start of therapy. Based on their genotype or phenotype, these three patients received initially reduced fluoropyrimidine dosages of 50%. They experienced limited and reversible toxicity (CTC-AE grades 0–2). The dose of one patient was increased to 70% in the second treatment cycle, after which CTC-AE grade 3 toxicity occurred.

Four of the seven case patients received a full dose, since their genotype was unknown prior to the start of therapy. These patients all experienced severe toxicity (CTC-AE grades 3–5), and three of them were admitted to the hospital for 7–14 days. An overview of cases, including the toxicity, is shown in Table 3.

### 3.3. Frequencies of Compound Heterozygous DPYD Carriers without Phasing Information

The ExAC and gnomAD databases revealed an average MAF for DPYD*2A, DPYD*13, c.1236G>A, and c.2846A>T of 0.55%, 0.03%, 1.43%, and 0.27%, respectively. MAFs for ExAC and gnomAD separately are summarized in Table 4. The probability of identifying a compound heterozygous *DPYD* patient for two variants according to these databases was ≤ 0.008%, as was calculated using frequencies of combinations of *DPYD* variants. Results for each combination of *DPYD* variants are shown in Table 5. With several million fluoropyrimidine users each year, thousands of patients worldwide are compound heterozygous for a subset of these four *DPYD* variants.

### 3.4. Frequencies of Compound Heterozygous DPYD Carriers with Phasing Information

In the GoNL database, genetic data from 496 subjects (fathers and mothers only) was reviewed. One subject was found who carried two *DPYD* variants. This subject was a carrier of the *DPYD* c.1236G>A and *DPYD* c.2846A>T variants, both of which were located on a single allele (in *cis*). Based upon the data in GoNL, the probability of having compound heterozygosity of the four *DPYD* variants is <0.2%.

In the 1000 Genomes database, data of 2513 individuals were available. After the selection of unique, unrelated individuals, 407 individuals remained. One subject was found who carried two *DPYD* variants. This subject was a carrier of *DPYD* c.1236G>A and *DPYD* c.2846A>T, both of which were located on different alleles (in *trans*). Based upon the data in 1000 Genomes, the probability of having compound heterozygosity of the four *DPYD* variants is <0.3%.

In the LUMC clinical genetics database (exome trios LUMC), the analysis was restricted to the children, since this would allow phasing. None of the 433 children carried more than one *DPYD* variant, thus compound heterozygosity in this database is <0.2%. 

Despite the low frequency, compound heterozygous patients were identified in all databases except the LUMC clinical genetics database. However, the low frequency did not allow to determine the probability of in *cis* or in *trans* phasing of variants in a patient.

## 4. Discussion

Prospective genotyping of *DPYD* variants followed by individual dose adjustments is increasingly applied as the standard of care for patients starting fluoropyrimidine therapy. Standard dose reductions from CPIC and DPWG guidelines cannot be applied in patients who carry more than one *DPYD* variant, as the phasing of the variants is unknown. Despite the low population frequency of < 0.2%, the absolute number of identified compound heterozygous patients will increase as the number of genotyped patients increases and the panel of tested variants is expanded. To the best of our knowledge, this is the first study that describes a case series of compound heterozygous *DPYD* variant allele carriers and investigates diagnostic and therapeutic strategies for these patients.

Our study shows the clinical need for further information on the genotype, as four patients were identified as compound heterozygous carriers retrospectively and all of them experienced severe toxicity. These compound heterozygous *DPYD* variant allele carriers have an increased risk of developing severe fluoropyrimidine-induced toxicity if dosages are not adequately adjusted. Previously, compound heterozygous patients have been described with severe or even lethal side effects after fluoropyrimidine treatment [39,40]. Three patients in this study were prospectively identified as compound heterozygous carriers, received initial dose reductions, and developed only mild toxicities. 

Out of the four patients for whom we were able to retrieve phasing information, three were in *trans* and one was in *cis* orientation. Data from publicly available databases also showed that both in *cis* and in *trans* orientations exist. However, the recently updated CPIC guidelines on *DPYD* assumes in *trans* phasing for compound heterozygous patients [20]. The DPWG guidelines do not mention phasing; however, the dosing recommendations of the DPWG use the GAS, a score based on the activity of individual alleles [19]. This implies the need for phasing information. The assumption of in *trans* phasing could result in the underdosing of patients with variants phased in *cis*, and thus exemplifies the need for the determination of the phasing of variants.

In this study, we looked at different diagnostic strategies to determine the phasing of *DPYD* variants in compound heterozygous patients. In four patients, the phasing of *DPYD* variants could be determined using one of three different molecular methods. These methods are in the early phases of development, not routinely available, quite expensive, and not always conclusive. For two of these techniques, patient RNA is used, which degrades quickly after the blood draw unless specifically designed blood tubes are used. Compound heterozygous patients are rare, yet here we describe seven patients heterozygous for multiple *DPYD* variants. A limitation of our study is that most patients were identified retrospectively and in different institutions. Because of this, not enough of or not the right material was available for analysis, thus not all genotyping techniques could be executed in each patient. For two samples, tests failed or produced inconclusive results. For this reason, a formal comparison of their suitability to identify phasing was not possible. However, of the three explored molecular methods, PacBio sequencing seems most promising. While phasing improved the prediction of DPD enzyme activity, patients with identical combinations of *DPYD* variants and identical phasing showed considerable differences in DPD enzyme activity, which could potentially limit the added value of the determination of the phasing of *DPYD* variants. However, larger numbers of compound heterozygous *DPYD* variant allele carriers would be necessary to draw a firm conclusion.

The measurement of DPD enzyme activity in PBMCs was used as a reference to assess DPD activity. The method is well-established, commonly available, and shows limited intra- and interpatient variability [27]. However, recently, differences in intrapatient variability in DPD enzyme activity related to circadian rhythm were shown [41], which can result in the under- or overestimation of DPD enzyme activity. In this study, we present one patient with extremely low DPD enzyme activity, which could possibly be influenced by the presence of severe neutropenia, as DPD activity is normally measured in mononuclear cells. Therefore, DPD enzyme activity can differ depending on the clinical condition of the patient and should thus be measured prior to treatment.

A major question is whether genotyping or phenotyping is the best method to determine DPD activity to guide fluoropyrimidine dosing in patients carrying multiple *DPYD* variants. Despite the low population frequency, we present seven patients carrying multiple *DPYD* variants, of which three received initially reduced fluoropyrimidine dosages. However, based on these data, it is not possible to determine if a dose recommendation based on phased genetic information or DPD enzyme activity measured in PBMCs is safer. In three out of four cases, differences were observed between the theoretically calculated DPD activity using genotyping or phenotyping. These differences would result in different dosing recommendations. For example, there is a considerable interpatient variability in DPD enzyme activity in carriers of the *DPYD* variant c.1236G>A/HapB3 [12]. Due to this variability, genetic dose recommendations are categorized (e.g., 25 or 50%) on the average of the phenotypes. This categorization could explain the observed dosing differences derived from genotyping and phenotyping. Other variants of *DPYD* currently not routinely tested for or variants in other genes, e.g., *MIR27A* [42], might also be involved in reducing DPD activity or explaining fluoropyrimidine-induced toxicity. DPD enzyme activity measurements are well-established, and additional molecular methods to resolve phasing are still in early phases of development. Therefore, in our opinion, the current therapeutic strategy for compound heterozygous *DPYD* variant allele carriers should be to determine initial dose reductions based on a DPD phenotyping test, for example, by measuring enzyme activity in PBMCs. Dosing could be adjusted by the treating physician in subsequent cycles based on observed severe toxicity (or lack thereof).

## 5. Conclusions

In conclusion, patients carrying multiple *DPYD* variants are at high risk of developing severe toxicity. Additional analyses are required to determine the correct dose of fluoropyrimidine treatment. In patients carrying multiple *DPYD* variants, we recommend that a DPD phenotyping assay be carried out to determine a safe starting dose. The dose could be titrated in subsequent cycles based on observed toxicity.

## Figures and Tables

**Table 1:** Characteristics of patient cases. Shown per patient are primary tumor, treatment, capecitabine dose, executed assays (genotype, dihydropyrimidine dehydrogenase (DPD) enzyme activity, and additional assays) information. Additional assays are droplet digital PCR, PacBio sequencing (Menlo Park, CA, USA), or an in-house developed technique. For the executed assays, it is shown whether these were executed prior to treatment (P) or retrospectively (R). *Abbreviations:* BC: breast cancer; CRC: colorectal cancer; CAP: capecitabine; RT: radiotherapy; OX: oxaliplatin; BEV: bevacizumab; bid: *bis in die*/twice a day.

Patient #	Primary Tumor	Treatment	Capecitabine Dose	Executed Assays
1	BC	CAP	1000 mg/m^2^/bid	Genotyping (R), DPD activity (R), in-house technique (R), droplet digital PCR (R)
2	BC	CAP	800 mg bid (50%)	Genotyping (P), DPD activity (R), in-house technique (R)
3	CRC	CAP + OX	900 mg bid (50%) ^1^	Genotyping (P), DPD activity (P), PacBio (R)
4	BC	CAP	1500 mg bid	Genotyping (R), DPD activity (R ^2^)
5	CRC	CAP + RT	800 mg bid (50%)	Genotyping (P + R ^3^), DPD activity (R ^4^), PacBio (R)
6	CRC	CAP + OX	1000 mg/m^2^/bid	Genotyping (R), DPD activity (R)
7	CRC	CAP + OX + BEV	1000 mg/m^2^/bid	Genotyping (R), DPD activity (R)

^1^ Increased to 70% in the second cycle; ^2^ during hospital admission; ^3^ DPYD*2A was prospectively identified, c.2846A>T was retrospectively identified; ^4^ during treatment.

**Table 2:** Dose advice for compound heterozygous *DPYD* variant allele carriers. Shown per patient are *DPYD* variants, phasing of the *DPYD* variants, GAS, retrospective DPWG dosing advice based on phasing, DPD enzyme activity, and percentage of DPD enzyme activity considered for dose advice. According to the DPWG guidelines [19], a gene activity score can be given to compound heterozygous patients when phasing is known. Fully functional/reduced functionality = gene activity score of 1.5; fully functional/inactive = gene activity score of 1; reduced functionality/reduced functionality = gene activity score of 1; reduced functionality/inactive = gene activity score of 0.5; inactive/inactive = gene activity score of 0. *Abbreviations:* DPD: dihydropyrimidine dehydrogenase; GAS: gene activity score; DPWG: Dutch Pharmacogenetic Working Group; X: could not be determined.

Patient #	*DPYD* Variants	Phasing	GAS [12]	DPWG Dose Advice (% of Regular Dose)	DPD Activity (nmol/(mg×h))	Percentage of DPD Activity ^1^
1	DPYD*2A + c.1236G>A	in *trans*	0.5	25%	0.9	9%
2	DPYD*2A + c.2846A>T	in *trans*	0.5	25%	6.0	60%
3	c.1236G>A + c.2846A>T	in *trans*	1	50%	4.5	45%
4	DPYD*2A + c.2846A>T	unknown	X	X	0.11	1%
5	DPYD*2A + c.2846A>T	in *cis*	1	50%	7.2	72%
6	DPYD*2A + c.1236G>A	unknown	X	X	3.8	38%
7	DPYD*2A + c.1236G>A	unknown	X	X	1.6	16%

^1^ The reference DPD activity ranges from 5.9–14 nmol/(mg×h) [28], and therefore the percentage of DPD activity can be calculated using the average of the reference (9.9 nmol/(mg×h). This percentage could be used as a percentage of the regular dose.

**Table 3:** Toxicity profiles of compound heterozygous *DPYD* variant allele carriers. Shown per patient are *DPYD* variants, fluoropyrimidine dose as a percentage of the regular dose, and experienced toxicity with this dose. All patients retrospectively identified as *DPYD* variants carrier received full doses and experienced severe (CTC-AE ≥ 3) toxicity. All patients prospectively identified as *DPYD* variant(s) carrier received dose reductions and experienced a maximum of CTC-AE grade 2 toxicity with the initial dose. *Abbreviations:* CTC-AE: Common Terminology Criteria for Adverse Events v4.03.

Patient #	*DPYD* variants	Dose (% of Regular Dose)	Toxicity (Maximal CTC Grade)
1	DPYD*2A + c.1236G>A	100%	4
2	DPYD*2A + c.2846A>T	50%	1–2
3	c.1236G>A + c.2846A>T	50% → 70%	0 (on 50% dose) → 3 (on 70% dose)
4	DPYD*2A + c.2846A>T	100%	5
5	DPYD*2A + c.2846A>T	50%	0
6	DPYD*2A + c.1236G>A	100%	4
7	DPYD*2A + c.1236G>A	100%	3

**Table 4:** MAF per database. Three databases (GoNL, 1000 Genomes, and exome trios LUMC) containing phased data were checked for four *DPYD* variants. Two large online databases (ExAC and gnomAD) were checked to identify the MAFs of the individual *DPYD* variants. For each *DPYD* variant, the genotype distribution and MAF are shown. *Abbreviations:* MAF: minor allele frequency; HW: homozygous wild-type; HE: heterozygous carrier; HM: homozygous carrier; GoNL: Genome of the Netherlands; ExAC: Exome Aggregation Consortium; gnomAD: Genome Aggregation Database.

	Variants	DPYD*2A (rs3918290)		DPYD*13 (rs55886062)		c.1236G>A (rs56038477)		c.2846A>T (rs67376798)	
Databases		HW/HE/HM	MAF	HW/HE/HM	MAF	HW/HE/HM	MAF	HW/HE/HM	MAF
GoNL		489/7/0	0.7%	494/2/0	0.2%	475/21/0	2.1%	490/6/0	0.6%
1000 Genomes		405/2/0	0.2%	406/1/0	0.1%	389/18/0	2.2%	403/4/0	0.5%
Exome Trios LUMC		946/15/0	0.8%	946/0/0	0.00%	946/46/0	2.3%	946/2/0	0.1%
ExAC		60,627/624/5	0.5%	60,320/42/0	0.03%	60,652/1808/27	1.5%	60,687/317/1	0.3%
gnomAD		138,489/1586/10	0.6%	138,166/83/0	0.03%	138,407/3841/39	1.4%	138,478/792/1	0.3%

**Table 5:** Calculated frequencies for compound heterozygous *DPYD* patients. Using the average MAFs of the ExAC and gnomAD databases (for DPYD*2A, DPYD*13, c.1236G>A, and c.2846A>T, these are 0.55%, 0.03%, 1.43%, and 0.27%, respectively), possible combinations for two out of four currently genotyped *DPYD* variants are shown. *Abbreviations:* MAF: minor allele frequency; ExAC: Exome Aggregation Consortium; gnomAD: Genome Aggregation Database.

Combination of *DPYD* Variants	Calculated Frequency
DPYD*2A + DPYD*13	0.0002%
DPYD*2A + c.1236G>A	0.008%
DPYD*2A + c.2846A>T	0.001%
DPYD*13 + c.1236G>A	0.0005%
DPYD*13 + c.2846A>T	0.0001%
c.1236G>A + c.2846A>T	0.004%

## Supplementary Materials

The following are available online at http://www.mdpi.com/2073-4425/9/12/585/s1; Description of patient cases (1 to 7) and Figure S1 illustration of molecular methods.
