# Supplementary material for: Diagnostic and Therapeutic Strategies for Fluoropyrimidine Treatment of Patients Carrying Multiple DPYD Variants
Source: Genes (Basel). 2018 Nov 28;9(12):585. doi: 10.3390/genes9120585 (PMC6316498; doi:10.3390/genes9120585)
Supplement: Supplementary file 1 [file genes-09-00585-s001.pdf]

Supplementary material

Patient cases

Patient 1. A 50 year old patient was diagnosed with breast cancer (cT3N3M0, triple negative), for which neo-adjuvant chemotherapy was started, followed by a breast saving surgery and axillary lymph node dissection (ypT1N1), and thereafter radiotherapy. Approximately a year later, lymph node metastases were found, for which capecitabine (1,000 mg/m^2 bid) was started. The patient experienced severe toxicity in the first cycle of capecitabine and was hospitalized for one week (allergic reaction -red swollen face-, complaints in the mouth, neutropenic fever, nausea, diarrhea, leukopenia CTC-AE grade 4). The patient was genotyped hereafter and turned out to be a carrier of *DPYD**2A and c.1236G>A. DPD enzyme activity was determined and was reported to be as low as 0.9 nmol/(mg*h) (normal range 5.9-14 nmol/(mg*h)). Internal evaluation using plasmids showed that these variants were both located at a different chromosome (in *trans*). Digital droplet PCR showed inconclusive results.

Patient 2. A 44 year old patient was diagnosed with breast cancer (cT1N1M0), for which the patient underwent surgery followed by radiotherapy and adjuvant chemotherapy (cyclophosphamide/methotrexate/5-FU dosed 600 mg/m^2 iv). Approximately seven years later, a relapse was discovered and one breast was removed followed by hormonal therapy (tamoxifen for three years, anastrozol for three years). Three years hereafter, palpable axillary lymph nodes were found, which turned out to be metastases of the tumor. TAC (docetaxel, doxorubicin, cyclophosphamide) cycles were started, however poorly tolerated and therefore cycles 2-6 were continued on 75% of the dose. Axillary radiotherapy was given hereafter. All was followed by letrozole. Then, metastases in the liver, bones and adrenal glands were found. Palliative chemotherapy with capecitabine (1,000 mg/m^2 bid) was started, for which *DPYD* genotyping was performed. She turned out to carry both *DPYD**2A and c.2846A>T. Taking this result into account and previous 5-FU combination therapy without any problems, capecitabine was started at 50% dose (800 mg twice daily). The therapy was discontinued due to side effects (hand-foot syndrome CTC-AE grade 1, diarrhea CTC-AE grade 1-2 and abdominal cramps CTC-AE grade 1-2). Internal evaluation using plasmids showed that the SNPs were both located at a different chromosome (in *trans*). For the purpose of this study, additional material was collected for DPD enzyme activity, which was low, but within normal range 6.0 nmol/(mg*h) (normal range 5.9-14 nmol/(mg*h).

Patient 3. A 61 year old patient was diagnosed with metastatic colorectal cancer (pT4N2M1). After a laparoscopic hemicolectomy was performed, chemotherapy with capecitabine and oxaliplatin was scheduled. The *DPYD* genotype was determined and two variants (c.2846A>T and c.1236G>A) were found. In addition to this, the DPD enzyme activity was measured, which was 4.5 nmol/(mg*h). The capecitabine dose was adjusted to ~50% (1,800

mg per day). Because of good tolerance, the capecitabine dose was increased to ~70% in the second cycle. Upon this increased dose, the patient developed thrombocytopenia CTC-AE grade 3 and complaints of anorexia, nausea and fatigue CTC-AE grade 1-2. In the third cycle, 57% dose was applied, resulting in a new thrombocytopenia CTC-AE grade 2. After these three cycles disease progression was noted and treatment was discontinued. For the purpose of this study, additional material was collected for the PacBio analysis and this patient carried both *DPYD* variants on different alleles (in *trans*). (Also described by Henricks *et al.* [1]).

Patient 4. A 38 year old patient was diagnosed with breast cancer. After surgery (pT2N0) and hormonal therapy (tamoxifen for three years) disease progression was established, and other hormonal therapies (anastrozole for two years, exemestane for two years andfulvestrant) followed. Due to liver metastases treatment was continued with capecitabine (1,500 mg twice daily). Severe side effects (CTC-AE grade 3 oral mucositis) occurred after 7 days of treatment for which chemotherapy was discontinued immediately and the patient was admitted to the hospital. During two weeks of hospital admission severe thrombocytopenia (CTC-AE grade 3), neutropenia and leukopenia (both CTC-AE grade 4) occurred. The patient deteriorated (respiratory problems, multiple organ failure), treatment against side effects was stopped and the patient died. During hospital admission, DPD enzyme activity was measured, which was extremely low (0.11 nmol/(mg*h)). Also, the *DPYD* genotype was determined and two variants (*DPYD**2A and c.2846A>T) were found. No additional analyses for this study were performed for this patient.

Patient 5. A 57 year old patient was diagnosed with colorectal adenocarcinoma (T4N0) for which a right hemicolectomy and trans anal endoscopic microsurgery of the rectum were performed. Approximately one year later, recurrence of rectal carcinoma was discovered (T4N2). The patient was also diagnosed with prostate cancer around the same time. Chemo radiotherapy was planned, consisting of radiotherapy on the rectum (25x2Gy) and prostate (total of 78Gy) combined with capecitabine. The patient participated in a clinical trial (NCT00838370) for which prospective genotyping (*DPYD**2A) was performed [2]. The patient tested positive and treatment was adjusted on the second day to 50% of capecitabine dose (800 mg twice daily). No severe side effects occurred. During treatment, DPD enzyme activity was measured, and was within normal range (7.2 nmol/(mg*h)). After treatment the patient was genotyped retrospectively for additional *DPYD* variants and was also a carrier of the c.2846A>T variant. For the purpose of this study, extra material was collected for the PacBio analysis and this patient carried both *DPYD* variants on a single allele (in *cis*).

Patient 6. A 67 year old patient was diagnosed with metastasized colorectal adenocarcinoma and treated with capecitabine (1,000 mg/m^2 twice daily) and oxaliplatin. The patient experienced CTC-AE grade 4 neutropenia, thrombocytopenia and leukopenia, CTC-AE grade 3 nausea, vomiting, diarrhea, stomatitis and anorexia. Toxicity resolved after

continuing with an unknown dose reduction. The DPD enzyme activity and *DPYD* genotype were determined after toxicity was resolved. A reduced enzyme activity of 3.8 nmol/(mg*h) and two variants (*DPYD**2A and c.1236G>A/c.1129-5923C>G) were found. Approximately a year later, the patient was again treated with the unknown reduced capecitabine dose and oxaliplatin, which was stopped after six cycles due to toxicity. No additional analyses for this study were performed for this patient.

<u>Patient 7.</u> A 61 year old patient was diagnosed with disseminated colorectal cancer (pT4N1M1) who underwent surgical hemicolectomy. Palliative chemotherapy consisting of capecitabine (1,000 mg/m^2 twice daily, day 1-14), oxaliplatin (130 mg/m^2, day 1) and bevacizumab (7.5 mg/kg, day 1) was started. After 11 days of chemotherapy, the patient was admitted to the hospital with CTC-AE grade 3 diarrhea and nausea with vomiting, and CTC-AE grade 2 fever. Capecitabine was stopped immediately. Loperamide therapy was started but the diarrhea persisted. The patient was discharged from the hospital after 13 days. The measured DPD enzyme activity was low (1.6 nmol/(mg*h)), and *DPYD* genotype was *DPYD**2A and c.1236G>A/c.1129-5923C>G, both measured after therapy. No additional analyses for this study were performed for this patient.

Figure S1:

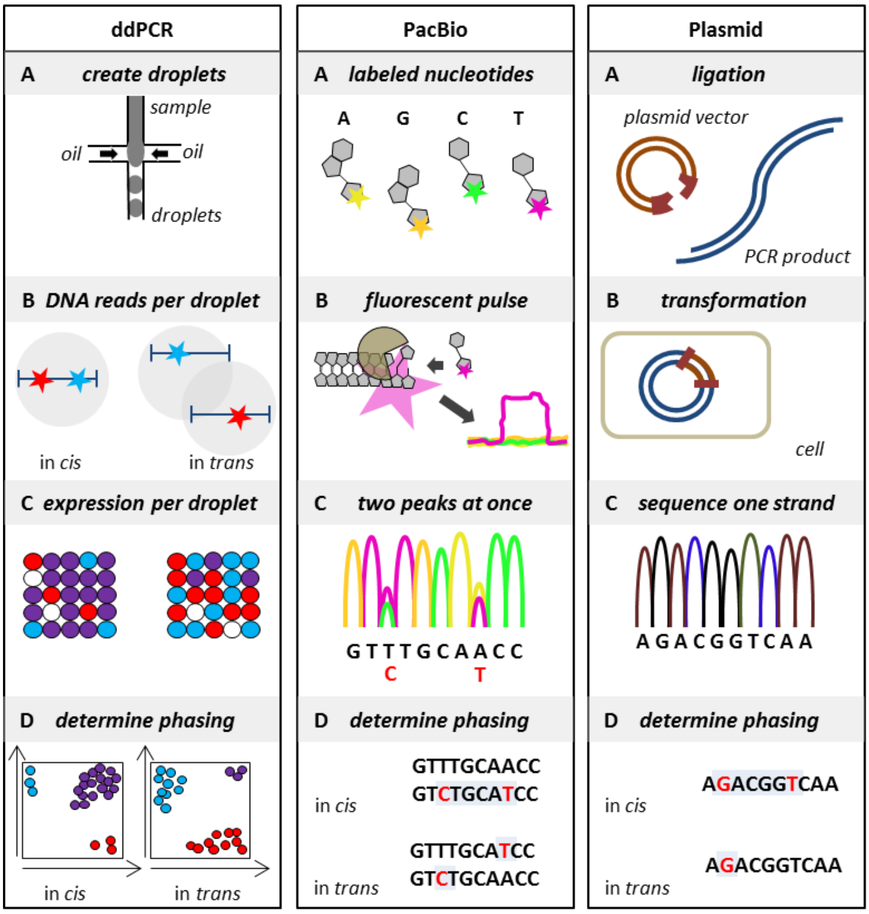

Illustration of molecular methods

ddPCR: Droplet Digital PCR (ddPCR) [3] is a method based on water-oil emulsion droplet technology. DNA isolation is performed using MagNA Pure Compact Nucleic Acid Isolation Kit I (Hoffmann-La Roche, Basel Switzerland). A DNA sample is fragmented into tens of thousands of droplets **(A)** and PCR amplification of DNA is present in each droplet **(B)**. PCR is based on standard TaqMan probe-based assays. In this study, we used FAM and HEX fluorescent labels, shown in red and blue (primers and probes used are; F-primer: CTGATCTTCATCTTCATTCC, R-primer: AGGTGGGAGAATTGTTGCTAT, probe: HEX-CCAGTTTCATCTTGTTCTGTCCGAACAA-BHQ, F-primer: TCACTGAACTAAAGGCTGA, R-primer: CAACTTATGCCAATTCTCTTG, probe: FAM-CTTTCCAGACAACATAAGTGTGATTTAAC-BHQ). In patients with two variants on a single allele (in *cis*) most droplets will emit both fluorescent labels, resulting in a combined color purple, where in patients with two variants on different alleles (in *trans*) most droplets only emit one fluorescent label, red or blue, when the DNA strands are being amplified. All droplets are read out one by one **(C)** and in *cis* or in *trans* phasing can be determined **(D)**. ddPCR can be used for DNA samples and detect phasing of variants in up to 200 kb. For *DPYD,* combinations of *DPYD**2A+*DPYD**13 (66 kb distance) and *DPYD**2A+c.1236G>A (124 kb distance) can be determined using ddPCR.

PacBio: Pacific Biosciences RSII (PacBio) [4] starts with RNA isolation from PAXgene tubes using RNeasy® Mini Kit (Qiagen, Hilden Germany) according to manufacturer's protocol. Then, cDNA is synthesized using SuperScript® III First-Strand Synthesis System for RT-PCR (Invitrogen, Carlsbad CA USA) using oligo-dT or *DPYD* gene specific reverse primers (F2: GTTTGCCAGAACCCAATAAAGA, F3: CGTCATTGTACTTGGAGCTGG, Rev: CCACAAAACCTGTATTACTGAATAA, Rev-comp: TTATTCAGTAATCAGGTTTTGTGG). cDNA is amplified using KAPA HiFi HotStart ReadyMixPCR Kit (Kapabiosystems, Wilmington MS USA). Amplicon preparation is executed according to PacBio® Procedure and Checklist - Amplicon Template Preparation and Sequencing. PacBio is based on single molecule real-time (SMRT) sequencing. On each of the four nucleotides different fluorophore labels are attached **(A)**, which will emit when the nucleotide is build-in, which is shown as a fluorescence pulse or color peak **(B)**. When a patient carries multiple variants, multiple fluorescent labels will be emitted at the same time, resulting in two color peaks simultaneously **(C)**. Variants can either be located on the same strand (in *cis*) or on different strands (in *trans*), determined by reading the strands **(D)**. The advantage of SMRT-sequencing is that longer read lengths of DNA or RNA are possible, therefore phasing of variants in the large *DPYD* gene can be determined.

Plasmid: cloning as described previously [5]. First, patient RNA was isolated using RNeasy® Mini Kit (Qiagen, Hilden Germany). With 500 ng RNA, cDNA was synthesized using 10 mol/µl oligodT primer in a 10.25 µl volume which was incubated for 10 minutes at 70 degrees Celsius. After cooling, 2 µl 0.1M DTT, 2 µl dNTPs (5 mM), 4 µl first strand buffer,

0.5 µl reverse transcriptase (RT), and 0.25 µl RNasin® (Invitrogen, Bleiswijk the Netherlands) was added and incubated for 1 hour at 37 degrees Celsius. Thereafter, a PCR was performed using Qiagens universal mastermix and primers approximately 500 nucleotides up or downstream of the variants (*DPYD**2A: ACCACCTCTGGCCCCATG, c1236G>A: GGTGGGAGAATTGTTGCTATG and c.2846A>T: GTAGCCAGAATCATTACAGG). Plasmids were created by ligation of the specific PCR products into pGEM-T Easy vector **(A)** (Promega, Leiden Netherlands) as follows: 0.5 µl pGEM-T Easy, 0.5 µl Ligase, 3 µl PCR product and 4 µl buffer was incubated for 2 hours at room temperature. Ligation mixture was transformed to competent E coli cells (JM109) **(B)** and plated on IPTG/Xgal (Promega, Leiden Netherlands) containing LB-ampicillin agar plates (Acumedia Neogen, Ayrshire UK). Plates were incubated overnight at 37 degrees Celsius. Next day, cells with successful insertions (resulting in white colonies) are grafted in 2 ml LB-ampicillin and shaken overnight at 37 degrees Celsius. Plasmid DNA was isolated using Miniprep Kit (Qiagen, Hilden Germany) and restriction enzyme EcoR1 as used to check for insertion of PCR product (approximately 1000 bp insert). Thereafter, sequencing was performed by Macrogen (Amsterdam, the Netherlands) using primers T7 (GTAATACGACTCACTATAGGGC) and SP6 (ATTTAGGTGACACTATAGAA) located on both sides of A-T ligation side **(C)**. Plasmids contain only one allele of the PCR product, thus combined sequence result of T7 and SP6 primers determines the haplotype. Thus, when only one variant was found, the unidentified variant is located on the other allele, and therefore phasing results are in *trans*. When both variants, or no variants were found, phasing results are in *cis* **(D)**.

References

1. Henricks, L.M.; Kienhuis, E.; de Man, F.M.; van der Veldt, A.A.M.; Hamberg, P.; van Kuilenburg, A.B.P.; van Schaik, R.H.N.; Lunenburg, C.A.T.C.; guchelaar, H.J.; Schellens, J.H.M., et al. Treatment algorithm for homozygous or compound heterozygous *DPYD* variant allele carriers with low dose capecitabine. *JCO Precis Oncol* **2017**. Published online October 6.
2. Deenen, M.J.; Meulendijks, D.; Cats, A.; Sechterberger, M.K.; Severens, J.L.; Boot, H.; Smits, P.H.; Rosing, H.; Mandigers, C.M.; Soesan, M., et al. Upfront Genotyping of *DPYD**2A to Individualize Fluoropyrimidine Therapy: A Safety and Cost Analysis. *J Clin. Oncol* **2016**, *34*, 227-234, doi:JCO.2015.63.1325 [pii];10.1200/JCO.2015.63.1325 [doi].
3. Regan, J.F.; Kamitaki, N.; Legler, T.; Cooper, S.; Klitgord, N.; Karlin-Neumann, G.; Wong, C.; Hodges, S.; Koehler, R.; Tzonev, S., et al. A rapid molecular approach for chromosomal phasing. *PloS one* **2015**, *10*, e0118270, doi:10.1371/journal.pone.0118270.
4. Buermans, H.P.; Vossen, R.H.; Anvar, S.Y.; Allard, W.G.; Guchelaar, H.J.; White, S.J.; den Dunnen, J.T.; Swen, J.J.; van der Straaten, T. Flexible and Scalable Full-Length *CYP2D6* Long Amplicon PacBio Sequencing. *Human mutation* **2017**, *38*, 310-316, doi:10.1002/humu.23166.
5. van der Straaten, T.; Swen, J.; Baak-Pablo, R.; Guchelaar, H.J. Use of plasmid-derived external quality control samples in pharmacogenetic testing. *Pharmacogenomics* **2008**, *9*, 1261-1266, doi:10.2217/14622416.9.9.1261.
